# Sarcoptic mange changes bacterial and fungal microbiota of bare-nosed wombats (*Vombatus ursinus*)

**DOI:** 10.1186/s13071-022-05452-y

**Published:** 2022-09-13

**Authors:** Christina Næsborg-Nielsen, Raphael Eisenhofer, Tamieka A. Fraser, Vicky Wilkinson, Christopher P. Burridge, Scott Carver

**Affiliations:** 1 Department of Biological Sciences, University of Tasmania, Private Bag 55, Hobart, TAS Australia; 2 Center for Evolutionary Hologenomics, GLOBE Institute, University of Copenhagen, 1353 Copenhagen, Denmark; 3 School of Biological Sciences, University of Adelaide, Adelaide, SA Australia; 4 Center for Animal Health Innovation, Faculty of Science, Health, Education and Engineering, University of the Sunshine Coast, Sippy Downs, QLD Australia

**Keywords:** Sarcoptic mange, *Sarcoptes scabiei*, Bare-nosed wombat, Microbiota, Dysbiosis, *Staphylococcus sciuri*, *Corynebacterium*, *Pseudogymnascus*, *Debaryomyces*

## Abstract

**Background:**

*Sarcoptes scabiei* is globally distributed and one of the most impactful mammalian ectoparasites. Sarcoptic mange, caused by infection with *S. scabiei*, causes disruption of the epidermis and its bacterial microbiota, but its effects on host fungal microbiota and on the microbiota of marsupials in general have not been studied. Here, we (i) examine bacterial and fungal microbiota changes associated with mange in wild bare-nosed wombats (BNWs) and (ii) evaluate whether opportunistic pathogens are potentiated by *S. scabiei* infection in this species.

**Methods:**

Using Amplicon Sequencing of the 16S rRNA and ITS2 rDNA genes, we detected skin microbiota changes of the bare-nosed wombat (*Vombatus ursinus*). We compared the alpha and beta diversity among healthy, moderate, and severe disease states using ANOVA and PERMANOVA with nesting. Lastly, we identified taxa that differed between disease states using analysis of composition of microbes (ANCOM) testing.

**Results:**

We detected significant changes in the microbial communities and diversity with mange in BNWs. Severely affected BNWs had lower amplicon sequence variant (ASV) richness compared to that of healthy individuals, and the microbial communities were significantly different between disease states with higher relative abundance of potentially pathogenic microbial taxa in mange-affected BNWs including *Staphylococcus sciuri*, *Corynebacterium* spp., *Brevibacterium* spp., *Brachybacterium* spp., and *Pseudogymnascus* spp. and *Debaryomyces* spp.

**Conclusion:**

This study represents the first investigation of microbial changes in association with sarcoptic mange in a marsupial host, as well as the first investigation of fungal microbial changes on the skin of any host suffering from sarcoptic mange. Our results are broadly consistent with bacterial microbiota changes observed in humans, pigs, canids, and Iberian ibex, suggesting the epidermal microbial impacts of mange may be generalisable across host species. We recommend that future studies investigating skin microbiota changes include both bacterial and fungal data to gain a more complete picture of the effects of sarcoptic mange.

**Graphical Abstract:**

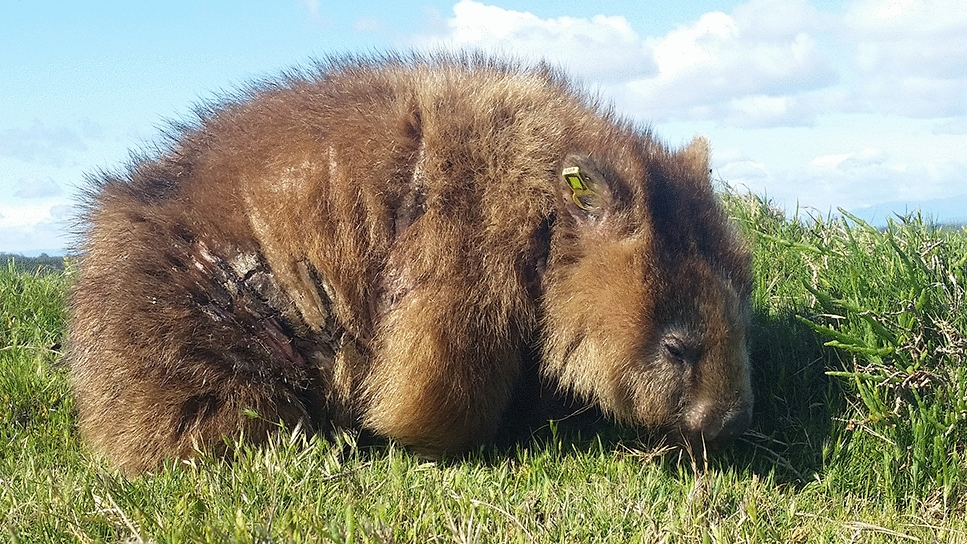

**Supplementary Information:**

The online version contains supplementary material available at 10.1186/s13071-022-05452-y.

## Introduction

The skin serves as protective physical barrier against pathogens and foreign substances and provides a habitat for a diverse range of bacteria, fungi, viruses, archaea and ectoparasites (e.g. mites, ticks, fleas) to inhabit [1, 2]. The composition and density of microbes define the skin microbiota, which in a healthy state may provide a level of protection against a range of threats to epidermal integrity [1]. For example, disruption of the skin microbiota can have implications for predisposing a host to infectious and inflammatory diseases [3], with these conditions typically leading to reduced diversity of microbial taxa and colonisation by potentially opportunistic pathogenic microbes that can further increase disease impacts.

*Sarcoptes scabiei* is one of the most impactful of mammalian ectoparasites: this skin-infecting mite is documented to affect nearly 150 species, occurs on every continent except Antarctica [4], and is considered a neglected tropical disease (NTD) in humans and an emerging panzootic in free-living wildlife [5–7]. Host species infested by *S. scabiei* tend to suffer either milder ‘ordinary mange/scabies’ or the more severe ‘crusted/Norwegian mange/scabies’, which is more commonly associated with mortality. Clinical signs of the disease range from mild erythaema to hyperkeratotic crusts, alopecia, and systemic disease with secondary infections [8–10] and potential death [11].

To date, studies of microbiota changes associated with mange have focused on humans, pigs, ibex, and canids [12–17]. Documented changes included reduced bacterial diversity and a higher abundance of *Staphylococcus* spp. and *Corynebacterium* in mange-affected individuals, which has been proposed to be due to excretions from the mite that inhibits the complement activation pathways [18] and the bacteria speculated to be the leading cause of secondary infections in multiple hosts [19–21]. However, it is unclear how consistent mange-microbiota relationships may be across other host taxa and geographic regions or whether inter-species and biotic/abiotic environmental differences might influence changes in microbiota. Furthermore, it is unknown how epidermal fungal microbes are impacted by mange, as fungi can trigger the innate and adaptive immune system, as observed in atopic dermatitis [22] and psoriasis [23, 24].

Sarcoptic mange was introduced to Australia by European settlers approximately 200 years ago [25] and has been confirmed in both marsupial and placental mammals [6]. Sarcoptic mange is the most impactful infectious disease of wombats, with the bare-nosed wombat (BNW, *Vombatus ursinus*) experiencing the most severe effects [26, 27]. Commonly, the BNW will mount a type IV hypersensitivity reaction to *S. scabiei* infection, but this immune response is insufficient to prevent mite proliferation, leading to host morbidity and eventually mortality [28], usually due to secondary infections. A range of studies have characterised clinical, behavioural, physiological, and immunological impacts of mange on BNWs [26, 28–30], but the epidermal microbial impacts in this host (or any marsupial) have not been investigated.

In this study, we (i) examine bacterial and fungal microbiota changes associated with mange in bare-nosed wombats; (ii) evaluate whether opportunistic pathogens are facilitated by *S. scabiei* infection in this species. We expect to find reduced bacterial diversity and a higher abundance of potentially pathogenic taxa, in line with what was reported in previous studies of other host species.

## Methods

### Sample collection and processing

Twenty-seven bare-nosed wombats were caught, anesthetised, and assessed using a standardised mange scoring system [31], and skin swabs and scrapings were collected in Narawntapu National Park, Tasmania, in 2015/2016 [32]. Skin swabs for microbiota analysis were collected using routine methodology [32]. Briefly, Catch-All sample collection swabs (Gene Target Solutions) were moistened with sterile saline and swabs were collected from the flanks (left and right sides), groin, abdomen, and toes in a 10 mm by 20 mm area, each site in a replicate of three (total *n* = 162). Swabs were stored at − 20 °C. Thirteen wombats that could be confidently assigned to a mange status were used for downstream statistical analysis; this reduced sample size provided more robustness to the severity categories. These individuals were classified as confidently healthy (observational score 0, PCR and total mite count 0, *n* = 6), mangy (observational score 2–3, mites present on PCR, *n* = 3), and severe mange (observational score > 3, mites present on PCR, *n* = 4) [32]. Additional file 1: Table S2 lists category criteria. The other 14 wombats could not be classified as confidently; see Additional file 1: Table S1.

### DNA extraction and amplicon sequencing

DNA was extracted with QIAmp Cell and Tissue extraction kit performed by the Garvan Institute of Medical Research including three No Template Controls (NTCs) for bacterial 16S rRNA gene and two NTCs for fungal ITS2 rDNA. All samples and Zymo DNA controls (16S = 3; ITS2 = 2, catalogue #D6306) were PCR-amplified and uniquely barcoded for Next-Generation Sequencing (NGS) using primers 515F/806r and fITS7/ITS4, for 16S and ITS, respectively. DNA sequencing was performed on an Illumina MiSeq (v2, 2 × 250 bp) at the Ramaciotti Center for Genomics (UNSW, Sydney). DNA sequencing data were processed and analysed using QIIME 2 2021.4 [33]. A Jupyter notebook containing all QIIME2 code is available for 16S and ITS [34]. The full dataset contained 9,415,845 raw reads across 168 samples (sample mean of 56,045.69) across 27 animals for 16S and 11,685,241 raw reads across 166 samples (mean of 70,393) across 27 animals for ITS2. The raw reads are available on NCBI.

Forward and reverse reads (R1 + R2) were imported into QIIME2 [33] and denoised using the dada2 [35] plugin with a trim length of 240 bp for 16S and a trim length of 0 for ITS2 due to the potential of removing important information as the ITS2 region is highly variable [36, 37]. Sequences were assigned taxonomy using the feature-classifier plugin (naïve Bayesian) on a pre-trained SILVA [38] 132 classifier [39] for 16S and on a trained UNITE [40] v8 10.05.2021 classifier for ITS and clustered at 99% similarity. A phylogenetic tree was created using the fragment-insertion plugin in combination with SEPP [41]. Contamination with exogenous DNA is always a risk during DNA extraction, sample preparation, and sequencing [42], which can produce false positives. To reduce this risk of sample contamination in our data, we explored the negative controls. Amplicon sequence variants (ASVs) that were detected in the negative controls and that had low frequency (< 10,000) in the overall frequency table were removed as they were likely the product of laboratory contamination, whereas ASVs present with high frequency (> 10,000) were kept in the analysis as these were most likely cross-well transfer from the true biological samples. Removed ASVs and their taxonomy can be found in Additional file 2: Dataset S1. The final analysed dataset comprised a total of 6,267,295 reads (12,086 unique ASVs) for 16S and 7,534,734 reads (3393 unique ASVs) for ITS2 from the 13 bare-nosed wombats (confidently healthy = 6, mangy = 3, severe mange = 4).

### Analyses

For both bacterial and fungal microbiota data, we calculated alpha diversity (observed OTUs) for a simple measure of species richness for each disease state and beta diversity (Bray-Curtis dissimilarity) for species abundance using the core-metrics-phylogenetic feature within the diversity plugin with a rarefaction depth of 13,084 and 5538 sequences per sample, respectively. Rarefaction depth was determined from the sample with the lowest number of reads after removing contaminants from the data. To test if and how statistically different bacteria and fungi were among mange classification groups we used a repeated measures ANOVA with wombat ID as the repeated measure (as multiple samples came from the same individual, *n* = 4 samples per individual, *n* = 2 samples per body site) and Tukey’s post-hoc comparisons to compare pairwise mange classification groups [43]. To test if and how much homogeneity of variance there were between severity groupings we used Levene test [44]. Microbial community structure (beta diversity) was visualised via non-metric multidimensional scaling (NMDS) of Bray-Curtis dissimilarity. For beta diversity we performed a PERMANOVA with wombat ID as a nested variable (the ‘adonis’ packages in R does not accommodate unbalanced random effects, but our preliminary analysis found nesting a sufficient alternative to accommodate the data structure). Furthermore, to evaluate the differential abundance of bacterial and fungal taxa across mange classification groups, we performed an analysis of composition of microbes (ANCOM) in QIIME2. The analysis required a composition artefact as input, which we created with additional filtering to remove taxonomic features that occurred in < 8 samples or frequencies < 50. The ASVs were then identified using NCBI BLASTn. We evaluated the effect of sex and body site variability in preliminary analyses using pairwise PERMANOVA and omitted these factors owing to sex having no effect on bacterial or fungal community structure and toes being statistically different from the rest of the body sites.

## Results

To examine the extent to which the skin microbiota is altered in sarcoptic mange-affected bare-nosed wombats, compared to healthy controls, we analysed a total of 6,267,295 reads (12,086 unique ASVs) for 16S and 7,534,734 reads (3393 unique ASVs) for ITS2 from the 13 bare-nosed wombats (confidently healthy = 6, mangy = 3, severe mange = 4). There was no difference between body site groin vs. left/right side (pairwise PERMANOVA; *F*_(3, 36)_ = 1.30,* P* = 0.100), whereas left/right sides vs. toes differed (PERMANOVA; *F*_(3, 36)_ = 1.71, *P* = 0.003) and there was a trend toward difference for groin vs. toes (PERMANOVA; *F*_(3, 36)_ = 1.29, *P* = 0.069). Owing to the distinction of toes from the other body regions, we excluded toes from the rest of the analysis.

### Sarcoptic mange reduces skin microbial diversity in BNWs

**Fig. 1:**
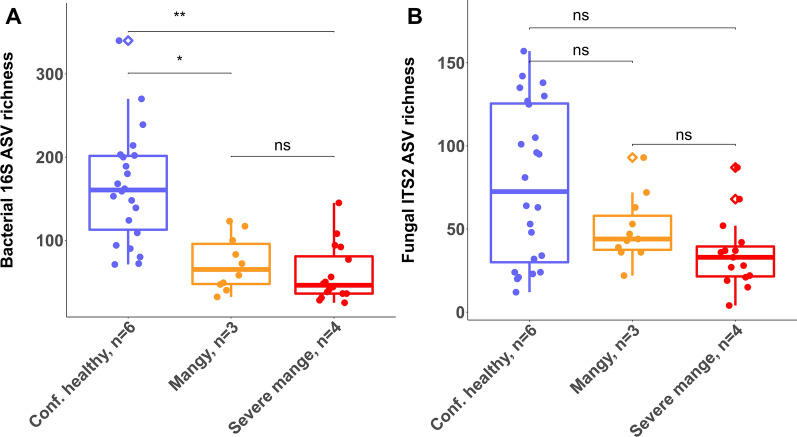
Wombat skin microbial diversity grouped by mange severity. ASV richness of 16S (**A**) and ITS2 (**B**). Results from Tukey’s HSD tests for pairwise comparison between mange groups are shown in brackets above boxplots; ns = not significantly different, ^*^ = post hoc *P* < 0.05, ^**^ = post hoc *P* < 0.01. Horizontal lines in the boxes represent the median values; the lower and upper bounds of the boxes represent 25th and 75th percentiles, respectively. Dots represent samples (several samples per wombat ID)

Microbial diversity differed significantly among severity groupings (repeated measures ANOVA: 16S *F*_(2, 10)_ = 12.79, *P* = 0.002; Fig. 1A). Bacterial diversity was greatest in the confidently healthy group of wombats, relative to the mange-affected groups, which did not differ from one another. In contrast, there was no significant difference in diversity among the ASVs for fungi (repeated measures ANOVA: ITS2 *F*_(2, 10)_ = 2.155, *P* = 0.167 Fig. 1B), although there was a trend for higher average fungal diversity in the confidently healthy group. Variance between severity groupings was slightly significant (homogeneity of variance, *F*_(2, 45)_ = 3.48, *P* = 0.03) in bacterial data with a significant difference between confidently healthy and severe mange. Variance was highly significant (homogeneity of variance, *F*_(2, 47)_ = 15.13, *P* > 0.0001) overall in fungal data with significant differences between confidently healthy and either of the two severity groupings.

**Fig. 2:**
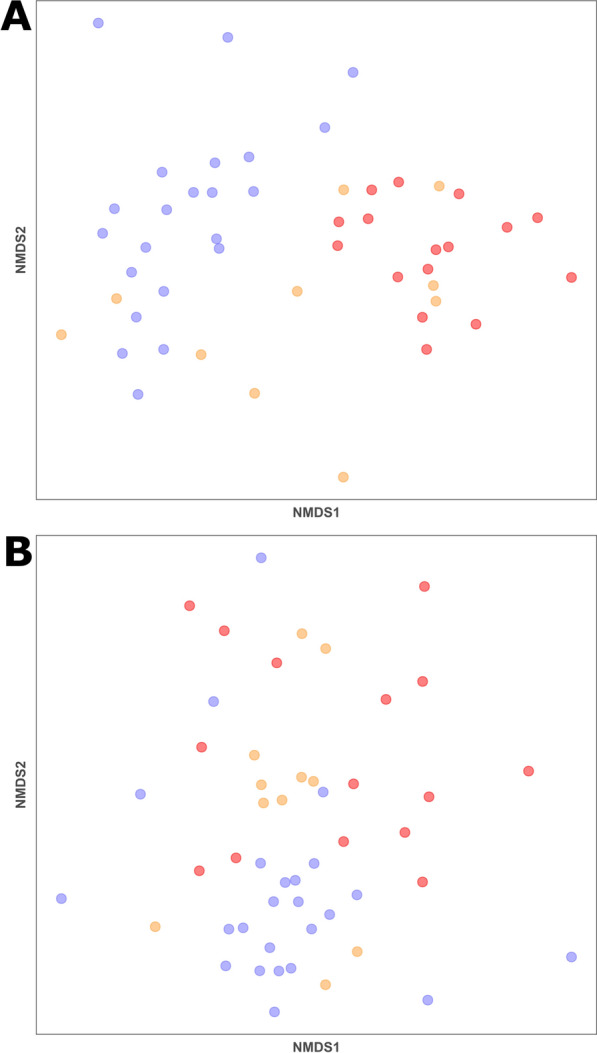
NMDS plots of Bray-Curtis Dissimilarity Index for **A** bacterial and **B** fungal microbial communities from bare-nosed wombats (BNW) with different mange severities. Blue = confidently healthy BNWs; orange = mangy BNWs; red = severe mange-affected BNWs. Each point represents a single sample

Analysis of the beta diversity showed that bacterial composition differed across the three groups (Fig. 2A, PERMANOVA, *F*_(10, 47)_ = 2.59, *P* > 0.001) and among each of the pairwise comparisons (*P* > 0.001). Fungal composition showed less distinct clustering based on mange severity (Fig. 2B), but differences were still statistically significant overall (PERMANOVA, *F*_(10, 49)_ = 2.16, *P* < 0.001) with distinction among each pairwise comparison. A fully annotated figure can be found in Additional file 1: Fig. S1.

### Sarcoptic mange influences skin microbiota composition in BNWs

**Fig. 3:**
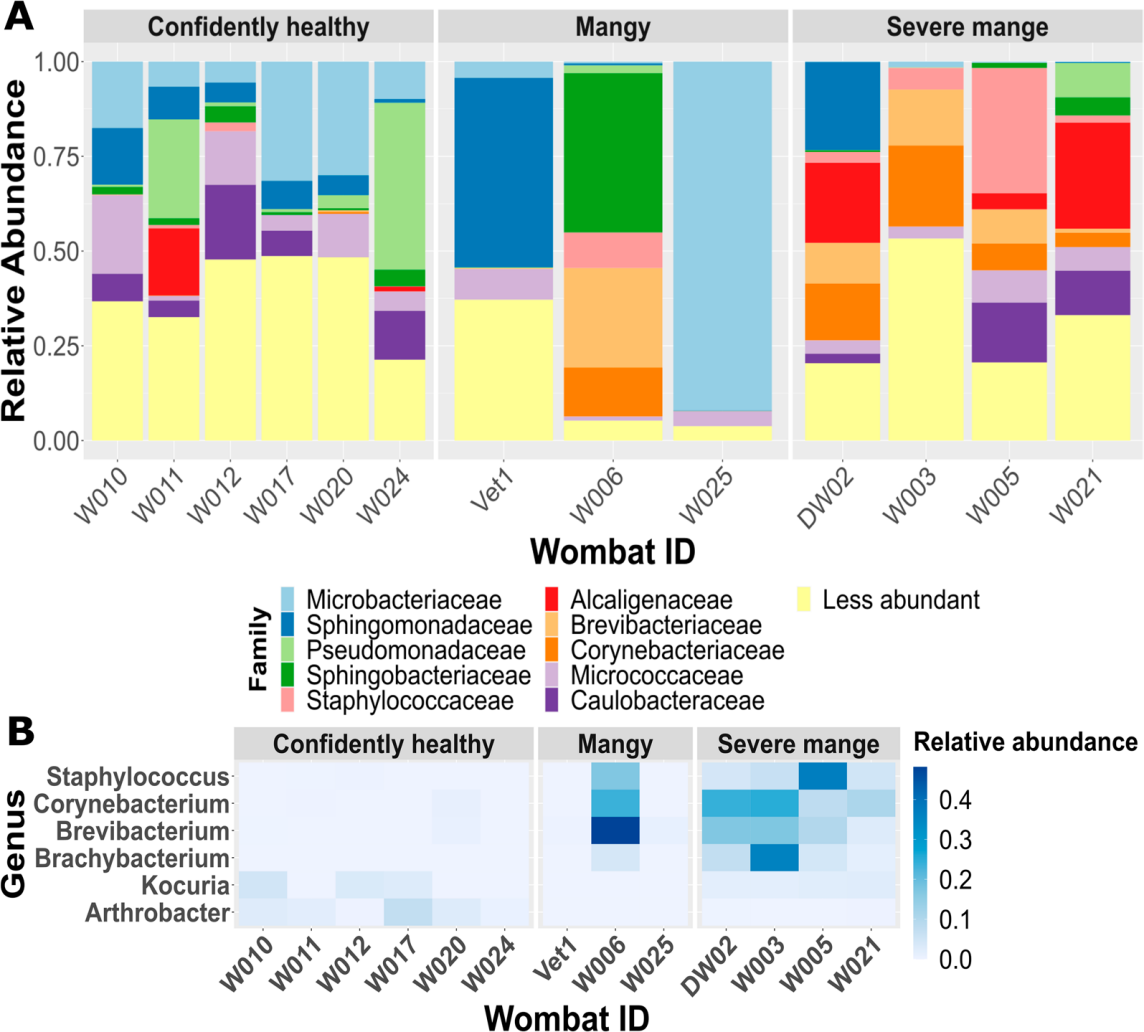
**A** Family-level microbial taxonomic bar chart of the ten most abundant families collapsed by wombat-id and sectioned into disease severity (confidently healthy, mangy, and severe mange); **B** notable bacterial taxa associated with mange severity. Observed changes in bacterial composition across severity groupings and individual bare-nosed wombats. Heatmap of selected bacterial genus

**Fig. 4:**
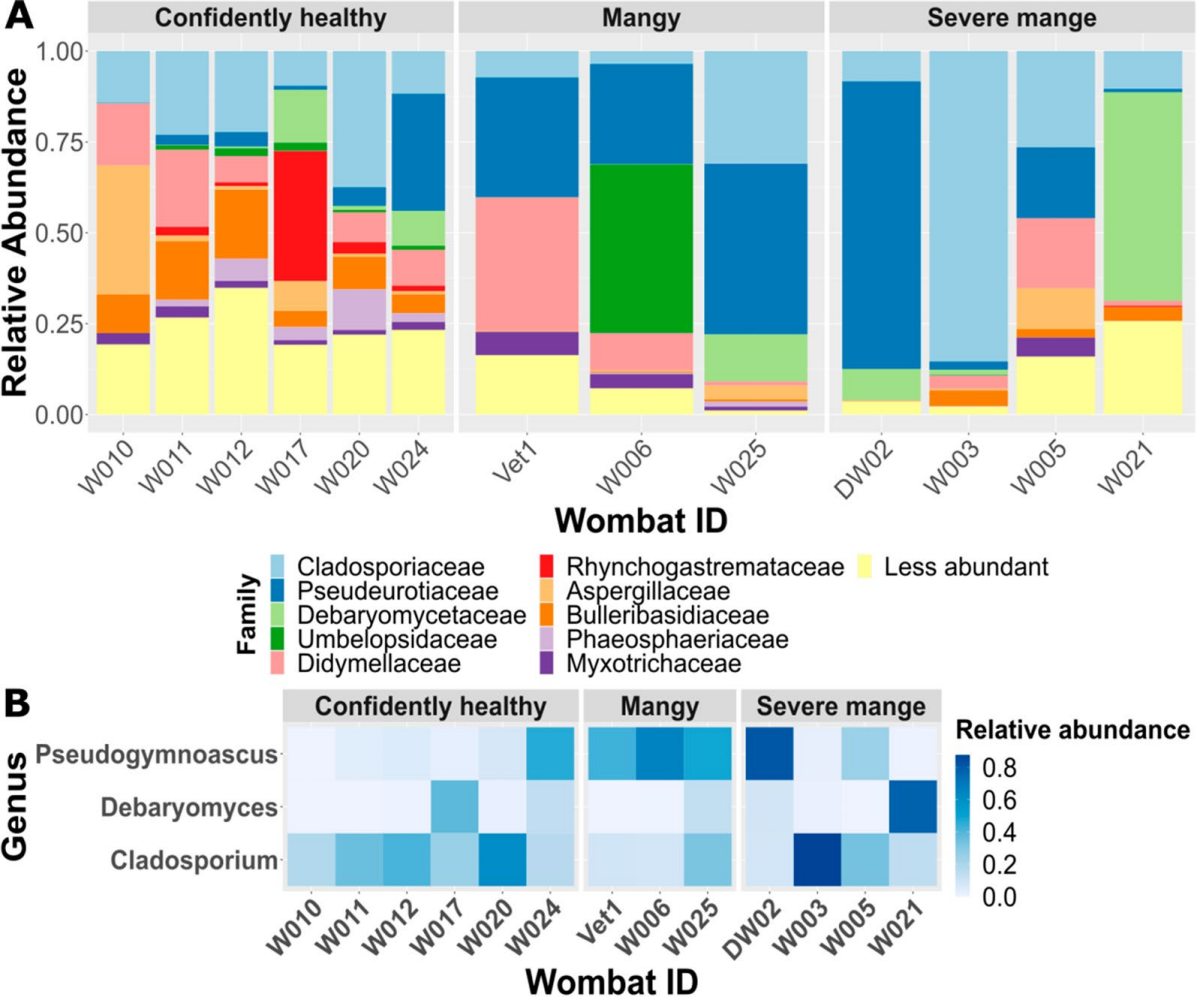
**A** Family-level fungal taxonomic bar chart of the ten most abundant families collapsed by wombat-id and sectioned into disease severity (confidently healthy, mangy, and severe mange); **B** notable fungal taxa associated with mange severity. Observed changes in fungal communities across severity groupings and individual bare-nosed wombats illustrated in a heatmap of selected fungal genus

The microbial phyla with the greatest relative abundance in BNW skin samples were Proteobacteria and Actinobacteria across all severity groupings. Ten microbial families accounted for approximately 90% of the skin microbiota (Fig. 3A). Variation between individuals was evident in both Fig. 3A and Fig. 4A; however, change in relative abundance of families can also be observed with disease severity. Healthy wombats displayed 854 bacterial genera, with fewer in mangy (54) and severely (232) mange-affected BNWs. Several distinct bacterial abundance changes were associated with mange severity based on differential abundance testing, including *Corynebacterium* spp. (*W* = 136, *clr* = 69.93), *Staphylococcus* spp. (*W* = 130, *clr* = 41.28), *Brachybacterium* spp. (*W* = 124, *clr* = 26.73), and *Brevibacterium* spp. (*W* = 129, *clr* = 33.71) with a higher abundance in mangy and severe mange groups, as well as *Kocuria* spp. (*W* = 129, *clr* = 87.92) and *Arthrobacter* spp. (*W* = 135, *clr* = 100.51) in healthy BNWs (Fig. 3B). Furthermore, NCBI BLASTn identified one of the ASVs with high sequence similarities to *Staphylococcus sciuri* (*W* = 126, *clr* = 32.11).

The fungal phyla with the greatest relative abundance in BNW skin samples were Ascomycota and Basidiomycota across all severity groupings. Ten microbial families accounted for approximately 90% of the skin microbiota (Fig. 4A). With disease progression, we observed decreased diversity of fungal genera when comparing healthy (651) relative to mangy (98) and severely affected (95) BNWs. We observed a general decrease of diversity with mange severity, as well as an increased relative abundance of *Pseudogymnoascus* spp., *Debaryomyces*, and *Cladosporium* associated with mange severity (Fig. 4B for quantification, Additional file 4: Dataset S3 for full taxa association), with exception in a few BNWs.

## Discussion

Sarcoptic mange is one of the most impactful of mammalian parasites globally, with detrimental effects on the health and welfare of animals [4], including the bare-nosed wombat [45]. The effects of mange on the skin microbiota have recently been investigated to identify potential microbes that causes secondary infections and patterns of skin microbiota disruption across infected individuals [12–17]. Our study characterised the bacterial and fungal changes in the skin of bare-nosed wombats at different stages of sarcoptic mange (healthy, mangy, and severe mange), finding comparable microbiota changes in this marsupial to other placental host species.

We found a general reduction in microbial richness and a change in microbial composition with mange disease severity. We identified bacterial genera such as *Brachybacterium*, *Brevibacterium, Corynebacterium*, and *Staphylococcus* in mange-affected individuals that were typically absent in healthy BNWs. The reduced skin bacterial diversity and increased relative abundance of *Corynebacterium* spp. and especially *Staphylococcus* spp. match patterns in humans [12, 13], North American canids [14], pigs [15], and Iberian ibex [16, 17]. *Staphylococcus* was present on almost all BNWs with moderate or severe mange and only in one sample in healthy wombats, suggesting an increased colonisation of *Staphylococcus* with disease. This corresponds to the patterns seen in crusted scabies in humans, where intensive scratching leads to secondary bacterial infections, most often caused by *Staphylococcus aureus* [12, 46] or *Streptococcus pyogenes* [20]. Infections such as these can lead to glomerulonephritis and rheumatic heart disease causing mortality [46]. ANCOM testing revealed the presence of *Staphylococcus sciuri* with disease progression. *Staphylococcus sciuri* is usually described as a commensal bacterium [47]; however, it has been known to cause disease in humans and other host species, such as fatal exudative epidermitis in piglets [48]. Whether *S. sciuri* is responsible for potential secondary infections is unknown, though future studies could investigate its pathogenic potential.

Microbial communities in crusts from pigs with severe mange were 80% *Corynebacterium* [15]. *Corynebacterium* naturally occurs in haematophagous arthropod gut microbiomes, and it is possible that mites could have brought *Corynebacterium* to the site of infection [15, 49]. Whether the *Corynebacterium* changes observed in the present study arose from *S. scabiei* intestine [50], environmental factors, or simply a response from the host (e.g. opportunistic colonisation) is unknown. Despite this lack of clarity, we observed an increase in the relative abundance of *Corynebacterium* as disease progressed from healthy to severe in the BNW suggesting a shift in the microbial balance with disease severity.

A limitation of our study is that the 16S V4 primer cannot definitively identify species [51], meaning we cannot say if the increases we are observing are colonisation of other opportunistic bacteria of the genus. However, this primer enables comparison to microbiome studies that have used this region. Thereby, we were not able to say whether any of the *Corynebacterium* spp. were of pathogenic nature; however, there are known pathogenic strains of the genus [3]. *Brevibacterium* has been documented as a non-pathogenic coloniser of human and southern hairy-nosed wombat (*Lasiorhinus latifrons*) skin [52]. However, in BNWs we observed an increase in its relative abundance with disease progression. The ANCOM test provided further evidence that the increases in both *Corynebacterium* and *Brevibacterium* were correlated with disease progression. *Kocuria*, identified using the ANCOM testing, was most likely part of the healthy skin microbiota with decreasing presence in mangy and severely impacted individuals. Furthermore, we want to caveat that inter-individual variation is to be expected even across healthy individuals [1, 53] as this is influenced by factors such as environment, time, and body site [54]. We attempted to control for body site variation by focusing on more microbially similar body sites such as the flanks, stomach, and groin with the rationale that samples from these regions reflected body areas routinely impacted by *S. scabiei*. The similarity was further evidenced by a non-significant difference between these body sites, contrary to toes.

Microorganisms such as fungi make major contributions to skin microbiota stability, health, and disease in humans [55, 56] and wildlife [57, 58]. However, they have never been studied during sarcoptic mange in any host species. We found that families such as Cladosporiaceae and Pseudeurotiaceae, and to some degree Debaryomycetaceae, had the highest relative abundance in mangy and severely mange-affected BNWs. These families were also present in healthy BNWs, albeit at a lower relative abundance due to the higher overall diversity. We observed no significant changes in ASV richness across severity groupings in fungal data; however, there were significant differences in variances and beta-diversity between severity groupings. Studies in humans have illustrated shifting epidermal fungal communities in patients suffering from chronic diseases, such as atopic dermatitis [59], or immunocompromised individuals [56]. These studies found increased fungal richness [54] with increasing abundance of opportunistic fungi and decreasing abundance of commensal fungi. Opportunistic fungi may colonise the skin with increasing disease severity, thereby outcompeting the rarer fungal species on the skin. Our intuition is that fungal pathogenesis in sarcoptic mange progression would be in line with what is observed in immunocompromised humans, i.e. more severely diseased individuals are more likely to contract fungal infections than healthy individuals. Additionally, bacterial and fungal microbiota are likely interacting, which might influence disease progression [60]. However, not enough is known about the shifting fungal composition of the skin to fully comprehend the role it might have on disease progression or if it is simply more of a marker of severe disease rather than contributing to the disease progression. This is an area for further research.

Due to the burrowing nature of the bare-nosed wombat, it is important to consider environmental influences in our data. *Cladosporium* is a common mould-type fungi isolated from soil and organic matter worldwide. *Cladosporium* was present on all bare-nosed wombats, though not detected in all samples, which is to be expected given sample and body site variability. It was more predominant in mangy and severely impacted wombats; however, that could simply be due to lower general microbial diversity in these two groups. In future research it would be beneficial to include soil swabs as positive environmental controls to potentially distinguish host and soil microbiota. *Pseudogymnoascus* is a widespread fungal genus of the Ascomycota, also often detected in soil. *Pseudogymnoascus* can breakdown keratin, causing skin infections in hosts [61], exemplified by *P. destructans* causing White Nose Syndrome (WNS) in North American bats. Our analyses suggested six different *Pseudogymnoascus* ASVs in the data. *Pseudogymnoascus* were present on the skin of all BNWs although it was likely to be more abundant on mangy or severely affected wombats. Whether the microbial changes observed are cause or consequence of disease progression is difficult to know. However, what is clear is that the observed changes are consistent across host species.

Some genera present in our data are commonly found as laboratory contaminants, such as *Sphingomonas* and *Pseudomonas* in bacterial data [42], and as environmental contaminants in fungal data, such as *Cladosporium* and *Penicillium* [62]. We have avoided conclusions pertaining to these genera. Given that *Pseudomonas* does not fluctuate across severity categories, its role as a common contaminant is supported by our dataset (see Additional file 3: Dataset S2).

Comparisons with skin microbiota responses to mange reported for other species.

Through a Web of Science search, we identified articles reporting skin microbial changes associated with sarcoptic mange in different hosts: human, domestic pigs (*Sus domesticus*), Iberian ibex (*Capra pyrenaica*), and three wild canid species. Across studies, increased observation of *Staphylococcus* spp. was found in mange-affected individuals, as well as *Corynebacterium* spp. (although missing in Iberian ibex). Human [12, 13], Iberian ibex [16, 17], and pig [15] studies all identified *Staphylococcus aureus* with severe disease. Additionally, an Iberian ibex study [16] identified *Staphylococcus warneri* as well as *Staphylococcus chromogenes* as potential pathogenic bacteria with sarcoptic mange. A North American study [14] identified *Staphylococcus pseudintermedius* in infected canids, whereas we identified *Staphyloccus sciuri* in mange-affected BNWs. The variation in *Staphylococcus* species identified could reflect host or environmental differences, most likely the former. Neither the N. American canid study [14] nor the pig study [15] described a change or presence of *Pseudomonas aeruginosa* in line with what was observed in humans [13] and Iberian ibex [16]. Our study provided evidence of continuous presence of *Pseudomonas*, but without fluctuations in disease status, and we were unable to identify the species (full overview is listed in Table 1).

**Table 1:** Syntheses of bacterial microbiota taxa changes (increased and reduced) associated sarcoptic mange disease in bare-nosed wombats (this study), humans, canids, pigs, and Iberian ibex

	Bare-nosed wombats (this study)	Humans	N. American Canids [14]	Pigs [15]	Iberian ibex
↑	*Corynebacterium spp.*	*Corynebacterium* [12]^a^	*Corynebacterium spp*.	*Corynebacterium*	
	*Staphylococcus spp.* and *S. sciuri*	*Staphylococcus aureus*	*Staphylococcus spp. and S. pseudintermedius*	*Staphylococcus aureus* and *Staphylococcus chromogenes*	*Staphylococcu*s *aureus* [16, 17] and *Staphylococcus warneri* [16]
		*Pseudomonas aeruginosa and* Group A *Streptococcus*, [13]^a^			*Pseudomonas aeruginosa* [16]^a^
	*Brachybacterium* spp. *Brevibacterium spp.*				
↓	Microbial diversity	NA	Microbial diversity	Microbial diversity	NA

*NA* not mentioned

^a^No healthy controls included in the study

## Conclusion

Our study provides the first insight into the effect of mange on the marsupial skin microbiota. These findings provide novel insights into shifts of bacterial and fungal communities relative to disease progression. Overall, we found similarities between the impacts of mange on BNW skin microbiota and other host species (canids, humans, pig, ibex), suggesting a common host impact from this cosmopolitan disease. Insights into skin microbiota changes in mange-affected hosts can provide critical additional knowledge to future therapeutic strategies, e.g. the use of pro- or antibiotic treatments in combination with traditional treatments, to help improve recovery. Future research into the skin microbiota changes with recovery from mange after treatment would indicate if the use of anti- or probiotics is beneficial. Furthermore, the inclusion of more taxa in skin microbiota studies could help highlight important drivers of mange pathology as interactions between taxa could influence disease progression.

## Supplementary Information

**Additional file1: Table S1** Mange classification of *S. scabiei* infestation based on the observational score and PCR status/total mite score. **Table S2** Explanation of determination of severity categories based on observational score and if mites were present in the skin scraping from the bare-nosed wombat. **Figure S1** NMDS plots of Bray-Curtis Dissimilarity Index for bacterial and fungal microbial communities from bare-nosed wombats.

**Additional file2: Dataset S1** Removed ASVs and their taxonomy.

**Additional file3:** **Dataset S2** Full bacterial taxa association.

**Additional file4: Dataset S3** Full fungal taxa association.

## Data Availability

Raw data is available on request. Code for processing is freely available on GitHub under project name “2021_BNW_skin_microbiome”.

## Abbreviations

BNW: Bare-nosed wombats
NTD: Neglected tropical disease
NTC: No template control
NGS: Next-generation sequencing
ASV: Amplicon sequence variants
OUT: Operational taxonomic unit
NMDS: Non-metric multidimensional scaling
WNS: White nose syndrome
