# Supplementary material for: Sarcoptic mange changes bacterial and fungal microbiota of bare-nosed wombats (Vombatus ursinus)
Source: Parasit Vectors. 2022 Sep 13;15:323. doi: 10.1186/s13071-022-05452-y (PMC9472346; doi:10.1186/s13071-022-05452-y)
Supplement: Supplementary file 1 — Additional file1: Table S1 Mange classification of S. scabiei infestation based on the observational score and PCR status/total mite score. Table S2 Explanation of determination of severity categories based on observational score and if mites were present in the skin scraping from the bare-nosed wombat. Figure S1 NMDS plots of Bray-Curtis Dissimilarity Index for bacterial and fungal microbial communities from bare-nosed wombats. [file 13071_2022_5452_MOESM1_ESM.docx]

Table 1. Mange classification of S. scabiei infestation based on the observational score and PCR status/total mite score

| Wombat ID | Mange classification | Observational score | PCR status | total mite count |
| --- | --- | --- | --- | --- |
| DW02 | Severe mange | 4.78 | Positive | NA |
| Vet1 | Mangy | 2.57 | NA | NA |
| W003 | Severe mange | 8.6 | Positive | 13 |
| W005 | Severe mange | 3.9 | Positive | 13 |
| W006 | Mangy | 2.7 | Positive | 2 |
| W010 | Confidently healthy | 0 | Negative | 0 |
| W011 | Confidently healthy | 0 | Uncertain | 0 |
| W012 | Confidently healthy | 0 | Uncertain | 0 |
| W017 | Confidently healthy | 0 | Negative | 0 |
| W020 | Confidently healthy | 0 | Negative | 0 |
| W021 | Severe mange | 4.9 | Uncertain | 18 |
| W024 | Confidently healthy | 0 | Negative | 0 |
| W025 | Mangy | 2.14 | Negative | 0 |
| W001 | Observational healthy | 0 | NA | NA |
| W002 | Uncertain to low mange | 0.5 | Negative | 2 |
| W004 | Uncertain to low mange | 1.9 | Uncertain | 2 |
| W008 | Uncertain to low mange | 1.9 | Positive | 8 |
| W009 | Uncertain to low mange | 0.4 | Negative | 0 |
| W013 | Uncertain to low mange | 0.5 | Negative | 0 |
| W014 | Uncertain to low mange | 0.28 | Negative | 0 |
| W015 | Observational healthy | 0 | Negative | 1 |
| W016 | Uncertain to low mange | 1.43 | Positive | 11 |
| W018 | Uncertain to low mange | 0.14 | Negative | 1 |
| W019 | Uncertain to low mange | 0.64 | Negative | 0 |
| W022 | Uncertain to low mange | 0.57 | Negative | 1 |
| W023 | Uncertain to low mange | 0.07 | Negative | 3 |
| W027 | Uncertain to low mange | 1.14 | Uncertain | 1 |

Table 2. Explanation of determination of severity categories based on observational score and if mites were present in the skin scraping from the bare-nosed wombat.

| **Severity category** | **Observational score** | **Mites present** |
| --- | --- | --- |
| Confidently healthy | 0 | No |
| Observational healthy | 0 | Maybe |
| Uncertain to low mange | 0.1-2 | Maybe |
| Mangy | 2.1-3 | Maybe |
| Severe mange | >3 | Maybe |


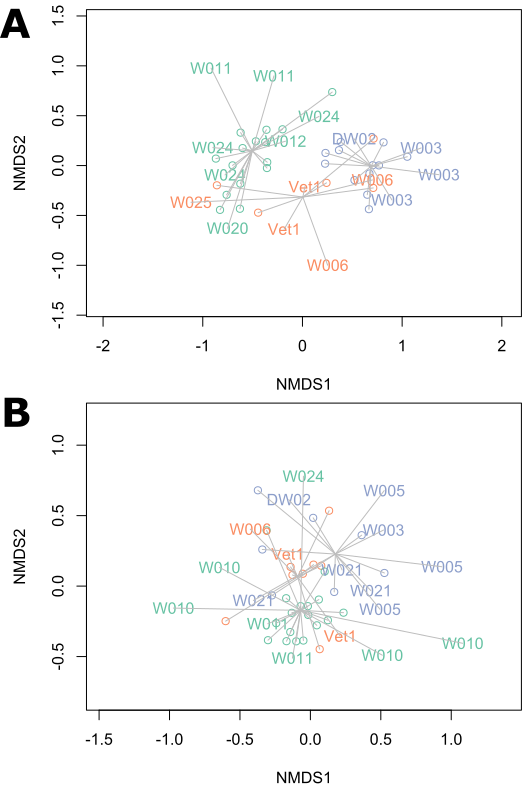


Figure 1. NMDS plots of Bray-Curtis Dissimilarity Index for A) bacterial and B) fungal microbial communities from bare-nosed wombats (BNW) with different mange severities.; Green = Confidently healthy BNWs; Red = Mangy BNWs; Blue = Severe mange affected BNWs. Each point represents a single sample.
